# Snf2 Family Gene Distribution in Higher Plant Genomes Reveals DRD1 Expansion and Diversification in the Tomato Genome

**DOI:** 10.1371/journal.pone.0081147

**Published:** 2013-11-28

**Authors:** Joachim W. Bargsten, Adam Folta, Ludmila Mlynárová, Jan-Peter Nap

**Affiliations:** 1 Plant Research International, Wageningen University and Research Centre, Wageningen, The Netherlands; 2 Netherlands Bioinformatics Centre (NBIC), Nijmegen, The Netherlands; 3 Laboratory for Plant Breeding, Wageningen University and Research Centre, Wageningen, The Netherlands; 4 Laboratory for Molecular Biology, Wageningen University and Research Centre, Wageningen, The Netherlands; 5 Centre for BioSystems Genomics 2012 (CBSG2012), Wageningen, The Netherlands; Nanjing Agricultural University, China

## Abstract

As part of large protein complexes, Snf2 family ATPases are responsible for energy supply during chromatin remodeling, but the precise mechanism of action of many of these proteins is largely unknown. They influence many processes in plants, such as the response to environmental stress. This analysis is the first comprehensive study of Snf2 family ATPases in plants. We here present a comparative analysis of 1159 candidate plant Snf2 genes in 33 complete and annotated plant genomes, including two green algae. The number of Snf2 ATPases shows considerable variation across plant genomes (17-63 genes). The DRD1, Rad5/16 and Snf2 subfamily members occur most often. Detailed analysis of the plant-specific DRD1 subfamily in related plant genomes shows the occurrence of a complex series of evolutionary events. Notably tomato carries unexpected gene expansions of DRD1 gene members. Most of these genes are expressed in tomato, although at low levels and with distinct tissue or organ specificity. In contrast, the Snf2 subfamily genes tend to be expressed constitutively in tomato. The results underpin and extend the Snf2 subfamily classification, which could help to determine the various functional roles of Snf2 ATPases and to target environmental stress tolerance and yield in future breeding.

## Introduction

In eukaryotes, genomic DNA is organized into chromatin, which is physically restricting the access of regulatory proteins to the genome [1]. The access to the genome can be changed by chromatin modifying activities, altering histone tails or the histone cores covalently; and chromatin remodeling activities, altering DNA–histone interactions non-covalently [1]. Both provide important epigenetic mechanisms to regulate gene expression [2]. The associated ATP-dependent changes in nucleosome organization catalyzed by Snf2-family ATPases accounts for a large part of chromatin remodeling activities [2]. 

Snf2 ATPases show broad functional diversity and are involved in a variety of genome-wide processes involving DNA, such as transcription, replication, repair and recombination. As ATPase they provide a motor that can translocate and move a complex directionally on double-stranded DNA [2]. In general, Snf2 family ATPases form large complexes with interacting partners [3], although few Snf2 family members can act alone [4,5]. Swapping the ATPase region of two different Snf2 family ATPases in different complexes can also exchange their functionality [6]. The Snf2 ATPases therefore shape the functionality of a complex. 

A first analysis of Snf2 family ATPases based on 30 sequences resulted in a classification of eight distinct subfamilies [7]. Snf2 family ATPases are characterized by seven helicase motifs [2,7,8]. The sequence spanning these motifs is called the Snf2 family ATPase region (Figure S1). The conserved ATPase region averages at about 400 amino acids [7] and is supposed to catalyze the translocase activity. A new survey of 1300 Snf2 family ATPases extended the classification to six groups (Snf2-like, Swr1-like, SSO1653-like, Rad54-like, Rad5/16-like and distantly-related Snf2 members) and 24 subfamilies [2]. The division into groups and subfamilies is based on phylogenetic analyses of the Snf2 family ATPase region. In many family members additional (accessory) domains are present, reflecting the sequence-based subfamily classification [3,8]. Not all subfamilies occur in every species or kingdom. An example is the DRD1 (defective in RNA-directed DNA methylation) subfamily occurring only in plant species [9,10]. 

In plants, functional annotation of Snf2 family members is most advanced in Arabidopsis. The Arabidopsis genome encodes 41 Snf2 family gene loci (http://www.chromdb.org; http://www.snf2.net). Encoded genes are distributed over six groups and 18 subfamilies. The specific function of the majority of the Snf2 proteins in plants is unknown [3], apart from the general contribution to DNA repair and recombination in development [2,11]. Different Snf2 ATPases, including members of the Snf2 and DRD1 subfamilies, have been shown to play a role in plant stress responses. Hence, the exploitation of such genes provides the basis for further functional characterization and could help develop plants that are better able to withstand environmental variation and/or (a)biotic stress. This may result in higher yields in less favorable environments. 

 We here present the first comprehensive analysis of Snf2 family members within the plant kingdom, to investigate phylogenetic relationships and infer putative specific functions of individual family members. Plant genomes show a high variability of the number of Snf2 genes, ranging from 17 to 63 members. The tomato (*S. lycopersicum*) genome shows gene expansions of the DRD1 subfamily with distinct expression patterns, suggesting further subfunctionalization of the duplicated members.

## Materials and Methods

### Genome sequence data, databases and software

Tomato (*S. lycopersicum*) assembly release 2.40 and iTAG annotation release 2.3 [12] were retrieved from the SGN network (http://www.solgenomics.net).The potato (*S. tuberosum* group Phureja DM1-3 516R44 (CIP801092)) genome assembly v3 and annotation v3.4 [13] were retrieved from the Potato Genome Sequencing Consortium (http://www.potatogenome.net). Where available, SGN Unigene builds (http://www.solgenomics.net; accessed on 7 October 2011) of other solanaceous species were used. Other green plant genome data were taken from Phytozome [14] (http://www.phytozome.net; version 7). The rice (*O. sativa*) annotation of Phytozome was enhanced by incorporating the annotation of the Rice Annotation Project Database [15,16]. In addition, protein sequences from ChromDB (http://chromdb.org; accessed on 7 October 2011), UniRef100 (http://www.uniprot.org; accessed on 7 October 2011) and RefSeq [17] (accessed on 7 October 2011) were used. Arabidopsis genome data were obtained from TAIR (http://www.arabidopsis.org/). Snf2 family analysis of Arabidopsis and rice was taken from the general Snf2 family protein resource (http://www.snf2.net/) for reference [8]. Taxonomy information was obtained from the Tree-of-Life project (http://tolweb.org/) and Phytozome. 

### Phylogenetic Analysis

Data preparation, conversion and filtering were performed with custom Perl scripts, BioPerl [18] and Bio::Phylo [19]. For the Snf2 gene calling in potato, potato protein sequences were determined by aligning all candidate Snf2 ATPase protein sequences against the potato genome using tBlastn [20] (E-value < 10). Hits were clustered into genomic regions with single linkage clustering (distance cut-off of 15kb) using C Clustering Library/Algorithm::Cluster [21]. Final gene models were predicted with Exonerate [22] using the parameters ‘--model protein2genome --showvulgar no --showalignment no --showtargetgff yes’ in the respective regions. Predicted potato gene models, unigenes, cDNAs and transcript sequences were translated using ESTScan2 [23] (additional parameter '-l 200') with the tomato hexamer frequency model obtained from SGN (http://www.solgenomics.net).

Domain detection was performed with HMMER v3.0 [24] and InterproScan [25] using Interpro Database version 35.0 (15 December 2011). Domain profiles were obtained from Pfam [26] and SMART [27]. A domain detection threshold of 1e-3 was used. It was adjusted with Arabidopsis as reference. To create an HMM model of the ATPase region, seed sequences were selected from UniProt, plant section, with the requirement of having the SNF2_N and Helicase_C domains present. Protein sequences smaller than 200 aa or with “putative”, “uncharacterized” or “predicted” in the description were excluded. The ATPase region was selected manually by identifying its conserved motifs Q-N (according to [8]) in the multiple alignment of the seed sequences. The model itself was trained with HMMER v3.0 [24], using hmmbuild with default parameters. A bitscore-based threshold of 200 was used to filter for Snf2 candidates. It was adjusted with Arabidopsis as reference.

Protein alignments were carried out with MAFFT v6.717b [28] using the E-INS-i mode with a maximum of 1000 iterations. Phylogenetic trees were estimated with RAxML v7.7.5 [29,30] using the fast bootstrapping mode and the JTT matrix model (parameters were ‘-x 12345 -p 12345 -f a -m PROTGAMMAJTTF’). 

Gene duplications and losses were evaluated with Notung [31]. Intrinsically disordered regions were analyzed with FoldIndex [32] using a score cut-off of -0.2. Phylogenetic trees were visualized with Dendroscope v3 [33] or E.T.E. [34].

### Expression data and analysis

Publicly available RNA-seq datasets from tomato (*Solanum lycopersicum* cv. Heinz 1706; data SRA049915) were retrieved from the SRA database (http://www.ncbi.nlm.nih.gov/sra). Sequence reads were mapped against the tomato reference genome (v. 2.40) with GSNAP [35]. The number of fragments per kb of exon per million fragments mapped (FPKM-values) were estimated for each gene model with cufflinks [36] on the basis of the iTAG 2.3 annotation and in-house enhanced gene models, where applicable. Conversions between SAM and BAM formatted alignments were performed with SAMtools [37]. Genes were categorized in three classes of expression: lowly expressed (FPKM ≤ 5), moderately expressed (5 < FPKM ≤ 200) and highly expressed (FPKM > 200). These categories are similar to a recent analysis of maize RNA-seq data [38], however without the more stringent cut-off proposed. For comparison, the cut-off based on the 95% confidence level was also used for analysis.

### RT-PCR analysis

Tomato cultivar Heinz plants were grown in a controlled greenhouse at 23°C in long-day conditions (16 h light/8 h darkness). Seedlings were grown on ½ MS (Murashige & Skoog) agar plates supplemented with 1% sucrose in a growing chamber at 25 °C in long-day conditions. Total RNA was isolated from 10-day-old seedlings, as well as from flowers, leaves and green mature fruits from greenhouse-grown plants using the E.Z.N.A.™ Plant RNA Mini Kit (Omega Bio-Tek, Inc., USA) followed by on column DNase treatment (Qiagen, RNase-free DNase Set). One microgram of RNA was used for cDNA synthesis using the iScript^TM^ cDNA Synthesis Kit (Bio-Rad Laboratories, Inc., USA) according to the recommendations of the manufacturer. Primers were designed with Primer3Plus (http://www.bioinformatics.nl/cgi-bin/primer3plus/primer3plus.cgi; [39]) and checked for uniqueness in the tomato genome v. 2.40/ ITAG annotation v. 2.3 with the short-sequence BLASTN search of the BLAST 2.2.22+ toolkit (http://blast.ncbi.nlm.nih.gov/Blast.cgi). Primers used are listed in Table S1. All primer pairs were validated by generating positive PCR reactions on genomic DNA. For RT-PCR, 2.5 µl of 10-times diluted cDNA was used. In all cases, actin was used as a reference gene [40]. The conditions used for all RT-PCR were: 95 °C for 4 min, followed by 25 to 35 cycles of 95 °C for 30 s, 60 °C for 30 s, 72 °C for 90 s and final extension at 72 °C for 7 min.

The activity of the primers was tested in a series of PCR reactions on genomic DNA with different concentrations of each primer. The concentration with highest band intensity was determined as the best primer concentration. The specificity of all primer pairs was established in a series of PCR reactions with tomato genomic DNA or cDNA to have only one single band of expected size (data not shown).

## Results

### Variable numbers of Snf2 family members in plant genomes

**Figure 1:**
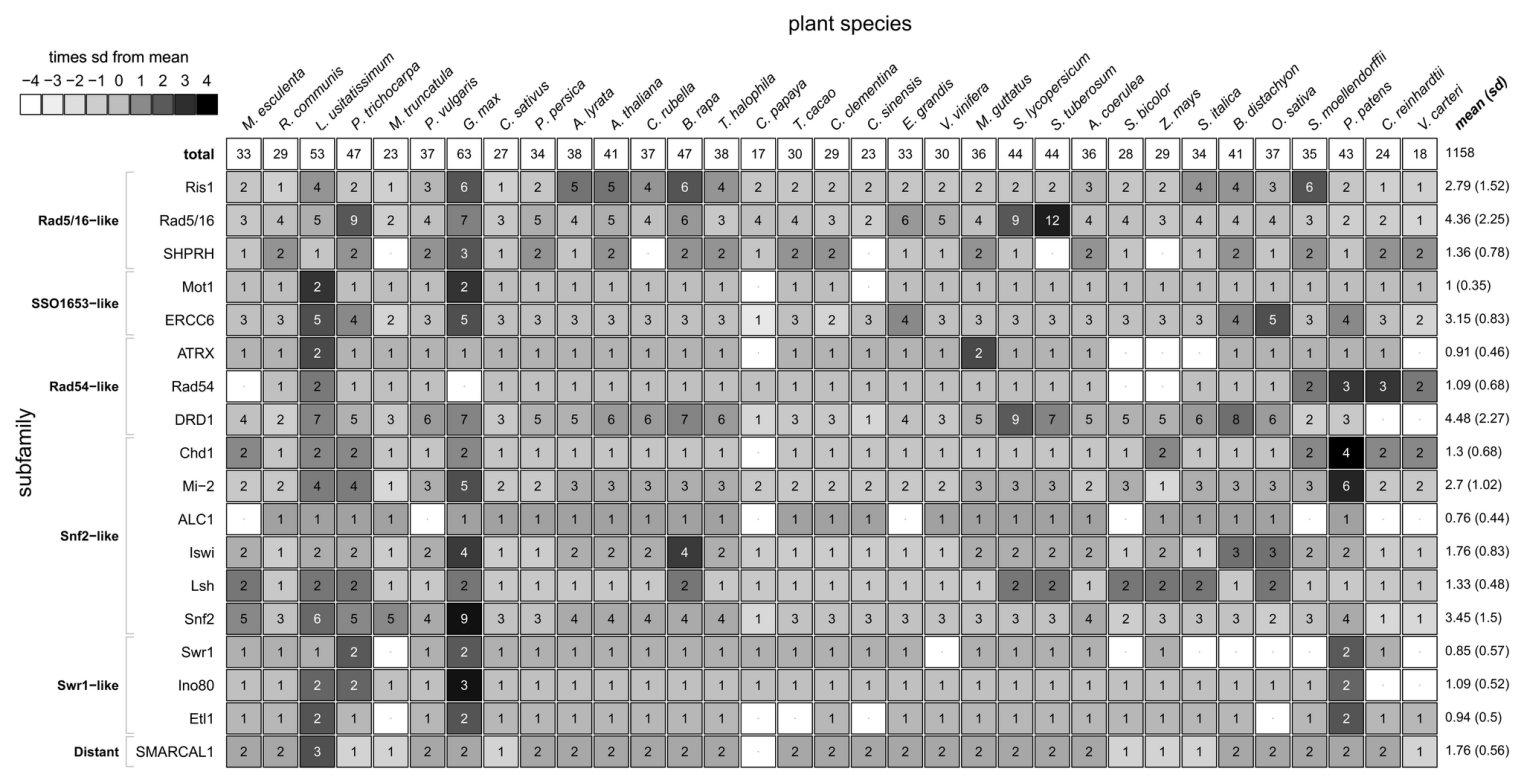
Distribution of Snf2 family members in plant genomes. Groupings and subfamilies on the left are named according to the Arabidopsis subfamily classification [3]. Species names on the top are organized on the basis of their phylogenetic relationship according to Phytozome [14]. Snf2 candidate member *Cre09.g390000.t1.1* (*Chlamydomonas reinhardtii*) could not be assigned to any subfamily and was excluded. Subfamily counts are shaded according to the deviation from the subfamily mean in standard deviations (sd). The total count is given on the top right cell. Mean and standard deviations per subfamily are indicated in the last column.

Snf2 family members in the predicted proteomes of 33 plant genomes including two green algae, were identified (Table S2). To prevent the inclusion of peptide fragments in the gene predictions, a cut-off of 200 amino acids (aa) was used, given that the conserved ATPase region has a length of about 400 aa [7]. All protein sequences longer than 200 aa were analyzed for the presence of the SNF2_N and Helicase_C domain. To be considered present, domains required a match in the protein sequence with an E-value smaller than 1e-3. Protein sequences containing at least one SNF2_N domain and one Helicase_C domain were listed as candidate Snf2 ATPase. To improve accuracy, a HMM model spanning the conserved ATPase region was created. The initial result set was filtered with this model and only candidates with a bitscore of at least 200 were used for further analyses. For Arabidopsis, all (41) previously known Snf2 genes (ChromDB; [41]) were identified (Figure 1). In total, 1159 family members were identified (Figure 1).

The total number of candidate Snf2 ATPases in plant genomes (Figure S2) shows considerable variation, ranging from 17-63 genes, with an interquartile range of 11, settled between 32 (Q1) and 43 (Q3). The papaya (*Carica papaya*) genome has only 17 candidate Snf2 family members, whereas in soybean (*Glycine max*, 63 members) and flax (*Linum usitatissimum*, 53 members) show an elevated number of family members. We identified 44 candidate Snf2 family members in the tomato genome (Figure 1), whereas the potato genome would carry only 23 candidate members that are also present in the official potato genome annotation. Given that both genomes are closely related in the *Solanaceae* genus, the surprising difference motivated an identification and re-calling of Snf2 genes in the potato genome. The re-calling identified 21 unannotated candidate Snf2 genes in the potato genome, in addition to the 23 from the first analysis. In other plant annotations, the number of potential Snf2 members was comparable between the genome annotation from Phytozome [14] and the re-calling (data not shown). Hence, all subsequent analyses were carried out with the set of 44 Snf2 family members in potato, the tomato annotation from ITAG and the annotation from Phytozome in all other cases. 

### Phylogenetic analysis

**Figure 2:**
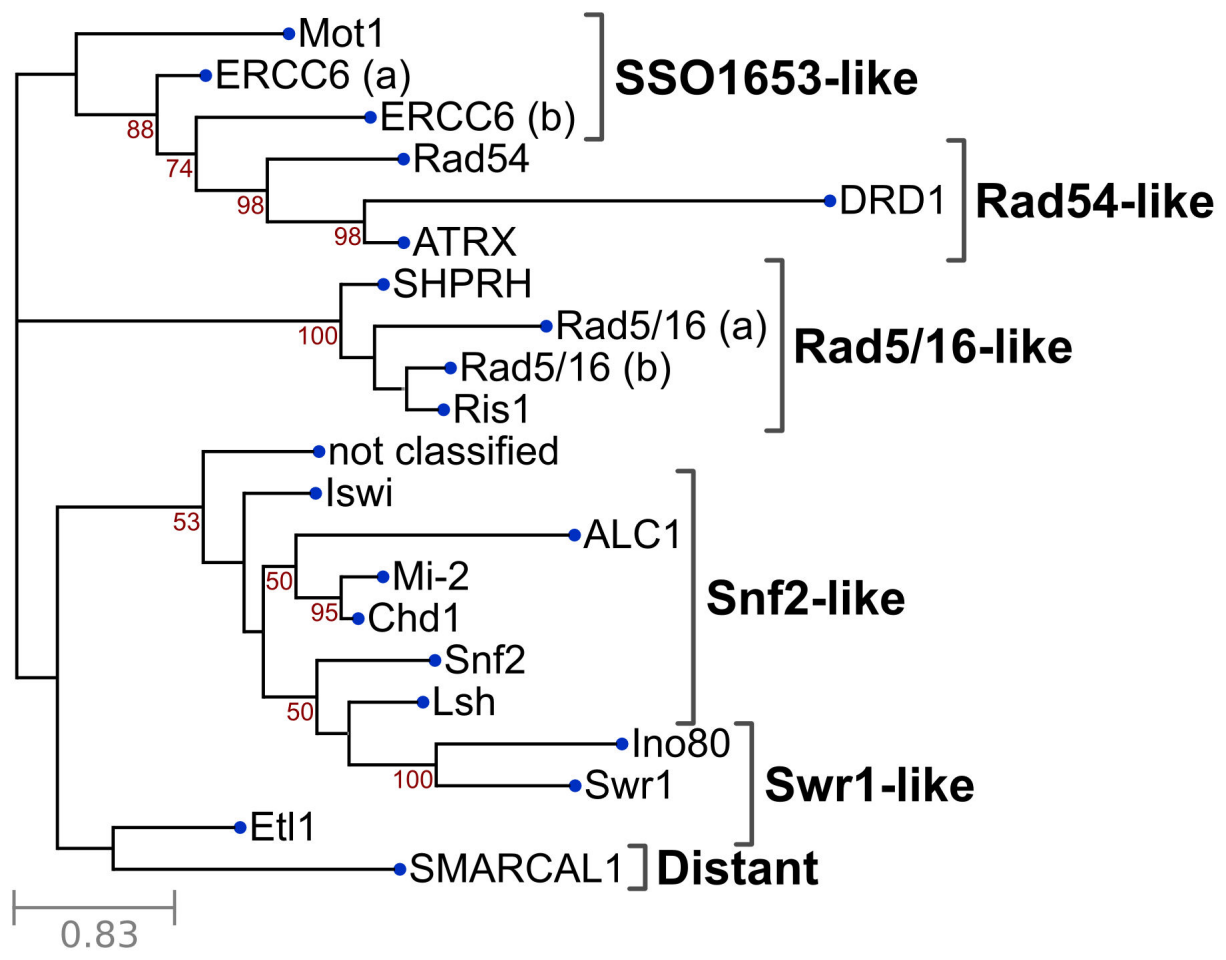
Unrooted phylogenetic tree of all candidate Snf2 genes in plant genomes. The full tree from which this subset was extracted is presented in Figure S3. The subfamily branches were collapsed to a single node that represents the first split that is part of the subfamily branch. Confidence values (50-100) are indicated at the relevant splits of the branches. The tree is based on 100 bootstrap replicates. The leaf tagged ‘not classified’ indicates candidate Snf2 members that are not part of a known subfamily, including *Cre09.g390000.t1.1* (*Chlamydomonas reinhardtii*) and members of sequence databases.

To infer evolutionary and potentially functional relationships of all plant candidate Snf2 genes, a phylogenetic tree was estimated on the basis of the conserved ATPase region of the protein sequence, including 30 aa flanking sequence on both sides to compensate for inaccuracies in domain prediction. To provide a more complete survey with focus on the Solanum genus, also transcriptome and unigene data (Table S2) were included. Each Snf2 subfamily was labeled according to the name of the Arabidopsis Snf2 subfamily in the relevant branch of the estimated tree. The unrooted tree summarizing the evolutionary relationships is presented in Figure 2. 

All 18 subfamilies identified are present in the tree and the overall tree topology of plant Snf2 genes is in agreement with earlier analyses [8], although members of the subfamilies Rad 5/16 and ERCC6 were distributed over two different branches. In green algae, only 3 of the 18 subfamilies are not present (DRD1, ALC1 and Ino80), suggesting a high conservation of Snf2 ATPases in the plant kingdom. The distribution of genes over the various Snf2 subfamilies per plant species is presented in Figure 1. For this estimation, only whole genome data were included. Half of the subfamilies occur in relatively small numbers (mean < 2), whereas 19 of 33 plant species miss one or more of these subfamilies. Four subfamilies (mean ≥ 3) are large: DRD1, Rad 5/16, Snf2 and ERCC6. Largest is the plant-specific DRD1 subfamily (148 members, mean 4.48), followed by the Rad 5/16 subfamily (144 members, mean 4.36) and the Snf2 subfamily (114 members, mean 3.45). Eight Snf2 candidate members originating form ChromDB, RefSeq and UniRef100 and the Snf2 candidate member Cre09.g390000.t1.*1* (*Chlamydomonas reinhardtii*) could not be assigned to any subfamily (not classified). These members were not taken into account. More plant genomes will have to be sequenced to ascertain whether the Snf2 family member distribution reflects any phylogenetic bias in genome sequencing.

### Snf2 family members involved in stress responses: DRD1 and Snf2

We focused further analyses on the two subfamilies reported to be connected to stress responses in plants, the DRD1 and Snf2 subfamilies [42–45] and on tomato and potato. Functional annotation of these subfamilies is guided by the functional information available for Arabidopsis genes.

### DRD1 subfamily

In Arabidopsis, the DRD1 subfamily has six members. Tomato has eleven members and potato seven. To characterize the phylogenetic relationships between the DRD1 subfamily members of plant species in the Asterid clade (potato, tomato and *Mimulus guttatus*) and Arabidopsis as model plant at a high resolution, the further analysis was focused on these four plants. According to the species tree (Figure S2), Mimulus is most close to the two solanaceous plants of interest. It has five DRD1 members. 

**Figure 3:**
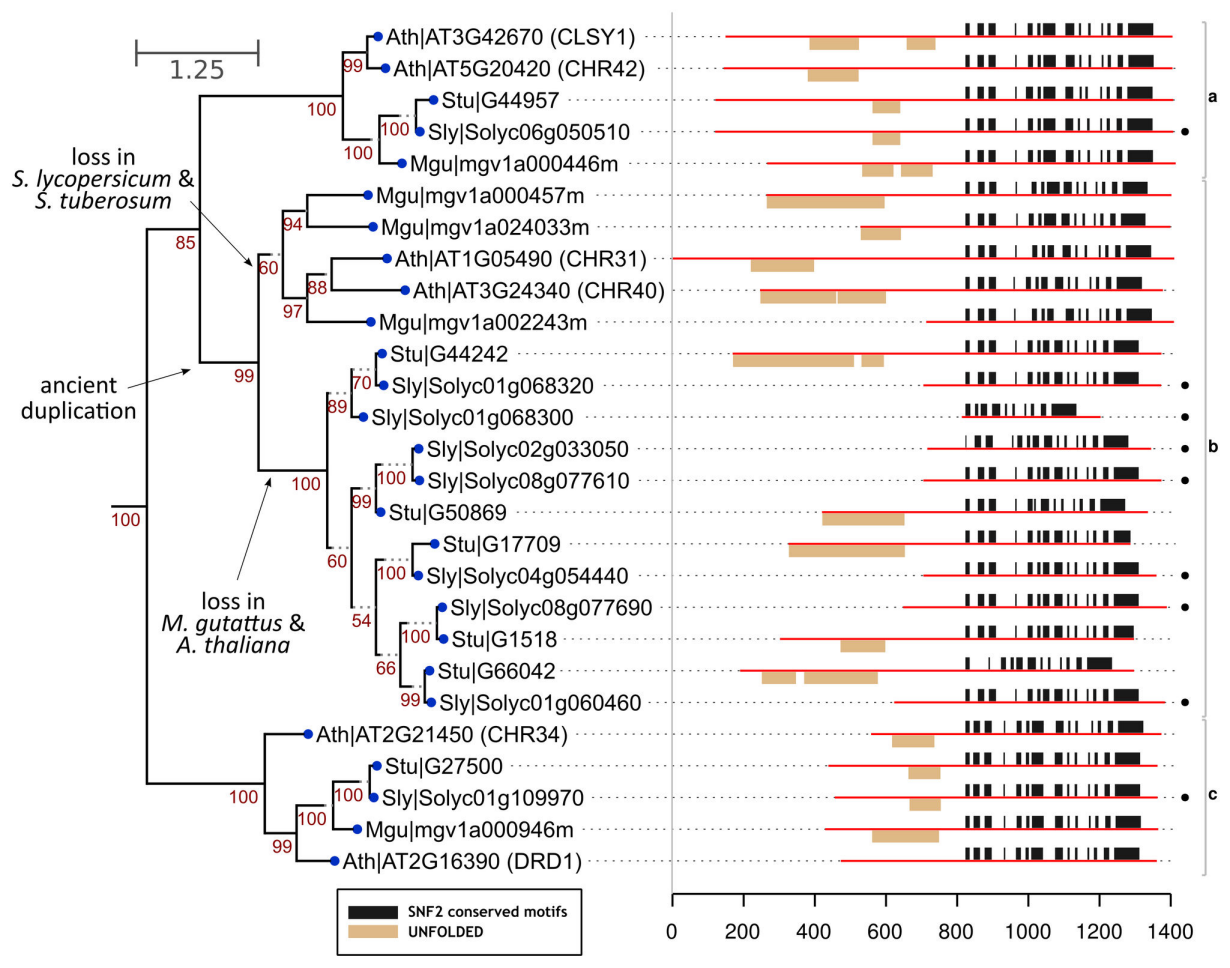
Analysis of the DRD1 subfamily in tomato, potato, Mimulus and Arabidopsis. The left side shows a detailed view of the DRD1 subfamily branch of an unrooted tree based on 1000 bootstraps of Snf2 data from *Arabidopsis thaliana* (Ath), *Mimulus guttatus* (Mgu), *Solanum lycopersicum* (Sly) and *Solanum tuberosum* (Stu). Confidence values (50-100) are given at the relevant branches of the tree. Identifiers give the name of the organism in three-letter abbreviations together with gene identifiers. The individual branches identified are indicated by letters in lowercase on the right side. To increase readability, some branch edges have been extended by dotted grey lines. These grey dotted lines are therefore not part of the estimated branch length. The right side shows structural elements (domains and unfolded regions) in the protein sequence of the DRD1 subfamily members in Arabidopsis, Mimulus, tomato and potato. Besides the ATPase region no other domains are present in these genes. A black dot at the right end of the figure indicates the expression of the respective gene in tomato based on the analysis of RNA-seq data.

In the unrooted phylogenetic tree based on the data from these four species (Figure 3), the DRD1 members could be grouped in three distinct branches, labeled a, b and c, each containing two Arabidopsis members. AtCHR42 and AtCLSY1 are in branch a, AtCHR31 and AtCHR40 in branch b, whereas AtDRD1 and AtCHR34 are in branch c. In all three branches, DRD1 members from tomato, potato and Mimulus are present. The tree shows that AtCHR42 and AtCSLY1 are in-paralogs [46] with one ortholog in tomato, potato and Mimulus (Figure 3; branch a). Likewise, AtDRD1 and CHR34 are in-paralogs with also one ortholog in tomato, potato and Mimulus (Figure 3, branch c). It is apparent from the tree that branch b is the most complex. In addition to the two members of Arabidopsis in branch b, Mimulus has 3, potato 7 and tomato 9 members. The number of members in branch c is relatively stable in other plant species, ranging from 1 to 3 (mean 1.49, sd 1, tomato and potato excluded). This indicates a relative expansion of DRD1 ATPases in the tomato and potato genomes. 

The potato/tomato members establish a separate sub-branch without members of either Arabidopsis or Mimulus suggesting independent evolution of DRD1 members in tomato and potato. Such evolution requires, the occurrence of a gene duplication in the common ancestor of all four species (labeled ‘ancient duplication’ in Figure 3), followed by independent gene losses in all four species. The high confidence value (99 from 100) for the ancient duplication supports this scenario. Also analysis with Notung [31] supports the mutual gene loss scenario (details not shown). The evolutionary history of solanaceous DRD1 genes suggests specific functions for such genes in tomato and potato. 

To infer potential functions of the DRD1 subfamily members, we investigated the presence of additional structural/functional elements in the protein sequences. The DRD1 subfamily members of the four species here investigated had no accessory domains (Figure 3). In many cases, the N-terminal region of DRD1 subfamily members shows a predicted disordered region. In Arabidopsis, this applies to all DRD1 subfamily members, except for the AtDRD1 protein (Figure 3).

### Snf2 subfamily

In Arabidopsis, the Snf2 subfamily has four members, while only three were found in tomato, potato and Mimulus. The tree estimated on data from these four species again shows three distinct branches (Figure S4), labeled a, b and c, respectively. The Arabidopsis genes AtCHR12 and AtCHR23 cluster together (Figure S4, branch a), in addition to single genes of the other species. It shows that AtCHR12 and AtCHR23 are in-paralogs with one ortholog in tomato, potato and Mimulus. The two Arabidopsis genes are likely to be the result of a gene duplication event specific to the Arabidopsis genus. The other Arabidopsis genes form one-to-one ortholog relationships with the respective tomato, potato and Mimulus genes (Figure S4). The evolutionary history of the Snf2 subfamily is therefore overall much less eventful than the history of the DRD1 subfamily. 

AtCHR12 and AtCHR23 (branch c) carry an unfolded region at the C-terminal end which is not present in any of the other members of the branch (Figure S4). The difference in length of the proteins in this subfamily is remarkable. Whereas branch a consists of relatively short proteins of approx. 1100 amino acids, branch b is characterized by very large proteins, the largest one (AtSYD) carrying 3574 amino acids. AtSYD has a considerably larger C-terminal end compared to all orthologs in its branch and compared to all members in the subfamily. Yet it only shows an unfolded region in the C-terminal end and no other functional or structural domains.

### Expression analysis of DRD1 and Snf2 subfamilies

Expression characteristics could also help elucidating the biological function of DRD1 and Snf2 subfamily members. We evaluated the expression profile of these genes in tomato public-domain RNA-seq libraries [12] for flowers, roots, leaves and various stages of fruit of tomato cv Heinz 1706 (Table S3). The FPKM-values of all libraries were calculated and visualized as heat map for the DRD1 and Snf2 subfamilies (Figure S5). All three Snf2 subfamily members of tomato are moderately expressed in the majority of the libraries analyzed. No tissue specificity and/or developmental control are apparent, suggesting a constitutive expression.

In contrast, expression of members of the DRD1 subfamily is more heterogeneous. The highest and most diversely expressed DRD1 subfamily genes are *Solyc01g109970* (branch c) and *Solyc06g050510* (branch a). *Solyc01g109970* is constitutively expressed in all libraries with FPKM values from 5 (leaves) to 37 (fully ripe fruit). Expression of the *Solyc06g050510* gene is similar, with the highest FPKM-value of 30 in roots, mature green fruits, immature fruits and 3-cm fruits. The lowest expression shows this gene in breaker and fully ripe fruits (FPKM around 7). The gene *Solyc01g068320* shows low specific expression in flower and flower bud tissue. The other 5 members that constitute the solanaceous-specific expansion of branch b in tomato show extremely low expression. 

**Figure 4:**
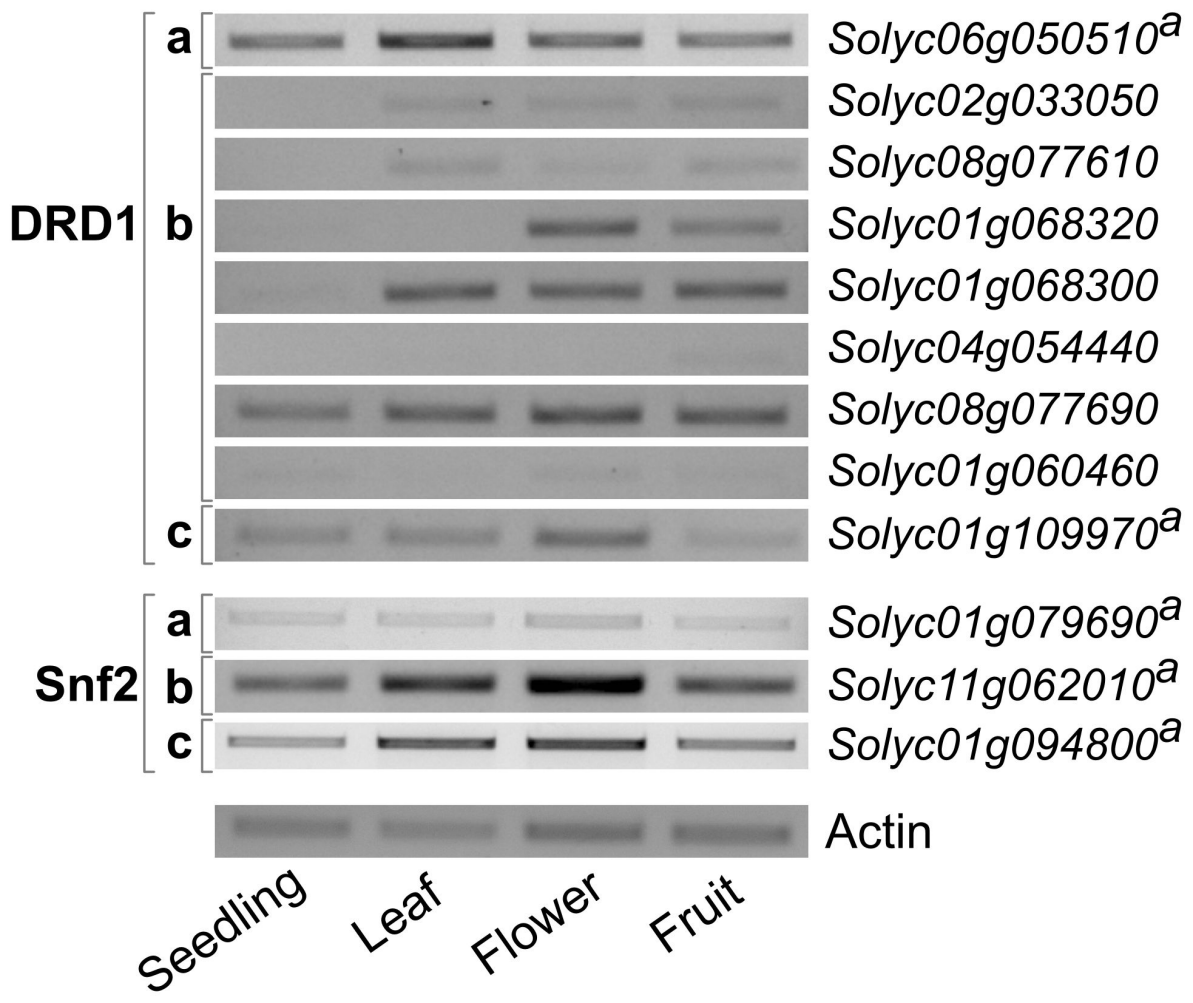
RT-PCR expression analysis of DRD1 and Snf2 subfamilies of tomato Snf2 ATPase genes. The tissue used is indicated on the x-axis. The individual genes are indicated of the right, the branches identified on the left. The expression of the actin gene (25 cycles) was used as control (lower panel). The number of PCR cycles used for the analysis of the individual gene was adjusted to generate a detectable amount of PCR product. For most of genes, 35 cycles were used. Genes marked with superscript a (^a^) were amplified with 29 cycles. For the actin gene 25 cycles were used.

To confirm these expression characteristics, semi-quantitative RT-PCR was performed on leaves, flowers and mature fruits. To be able to extend the analysis to early stages of plant development, 10-day-old *in vitro*-grown seedlings were included. RT-PCR analysis of the three Snf2 genes confirmed expression in all four tissues analyzed, in concordance with the RNA-seq analysis (Figure 4). It also largely confirmed the RNA-seq results of the DRD1 subfamily genes (Figure 4). *Solyc08g077690 i*s expressed in all tissues examined at the highest level shown by any member in this branch. Expression of *Solyc01g068320* is restricted to flower and fruit tissue, the latter at lower levels. For *Solyc01g068300* RT-PCR shows a relatively easily detectable product in all tissues except seedlings. Also expression of *Solyc02g033050, Solyc01g060460* and *Solyc08g077610* is detectable by RT-PCR in all tissues. However, the level of expression is low to very low, approaching the lower limit of reliable detection. Gene *Solyc04g054440* is very lowly expressed in possibly only fruits. The highly variable expression patterns of the various DRD1 subfamily genes indicate that the putative function of the encoded DRD1 proteins is likely to be subtle in terms of time or location. 

## Discussion

The Snf2 family of ATPases is a large family of chromatin remodeling enzymes that have versatile roles in a variety of fundamental processes in growth and development. In plants, little is known about the function of individual members of this family, although notably in Arabidopsis functional relationships with gene regulation, DNA recombination, DNA repair and stress tolerance have been reported [42,43,47]. Here, we present the first comprehensive comparative analysis of all Snf2 genes in 33 sequenced and annotated plant genomes, including two green algae. We have identified and analyzed 1159 potential candidate Snf2 family ATPases, of which all but one could be placed in previously established groups and subfamilies and represent genuine plant Snf2 genes. The variation in numbers of Snf2 genes is large, ranging from 17 in papaya to 63 in soybean. This suggests a broad functional diversification of this gene family in the plant kingdom. The high member counts in flax and soybean may originate from recent whole-genome duplications in both species [48,49]

Our results for rice show considerably more differences when compared to another recent study of Snf2 family genes [50], in which 39 putative Snf2 family genes are identified. The overall tree presented [50] does not seem to agree well with the subfamily classification. An example is a branch containing rice genes (Os02g0114000 (Snf2), Os01g0779400 (Ris1), Os05g0150300 (Iswi), Os05g0392400 (DRD1) and Os07g0497000 (Mi-2)) that are distributed in five different subfamilies according to our classification. Possible explanations for the differences are phylogenetic tree modeling based on the complete protein sequence rather than the conserved region, and/or the use of another rice annotation ([15]; http://rapdb.dna.affrc.go.jp).

Surprising sources of error in Snf2 family member identification are the publicly available genome assemblies and annotations. Our example in potato highlights the better performance of gene calling within a protein family opposed to automatic gene calling. Half of the Snf2 family members are absent from the current genome annotation of potato. Assembly and calling of Snf2 genes may be troublesome for the partly automated pipelines in place for overall genome assembly and annotation, despite manual curation effort. Here we show increased sensitivity of candidate Snf2 family gene identification by iterative rounds of homology-based gene prediction. This approach minimizes errors in the predicted coding region that would affect the multiple sequence alignment and phylogenetic reconstruction considerably. For Arabidopsis and rice, the plant species with the richest set of annotation and experimental data, inferred gene models were consistent with the currently available high-quality annotations (not shown). Therefore, the annotation of the potato Snf2 family is likely to have improved markedly with the homology-based prediction routine put in place and is recommended for future analyses. The accuracy of the prediction of the proper coding region is not likely to be improved with the help of (family-) specific gene models or better hexamer models. Such homology-based prediction will not safeguard against errors in assembly. 

Not anticipated from the earlier analyses of Snf2 family genes [8] is the relative expansion of the DRD1 (148 genes), Rad5/16 (144 genes) and Snf2 (114 genes) subfamilies in plant genomes. So far, members two of these subfamilies have been associated with environmental stress responses in Arabidopsis, possibly indicating the relative importance of chromatin remodeling in combatting environmental stress in plants. The most abundant subfamily, DRD1, has evolved from apparent non-existence in non-plant species (www.snf2.net) and lower plants, such as *Volvox carteri* and *Chlamydomonas reinhardtii*, to the largest and most diverse subfamily in current-day higher plants. It indicates that the DRD1 protein has become an important and possibly diversified asset in the regulation of plant growth and development. Within the expanded DRD1 subfamily, tomato has one of the highest member count of all genomes analyzed, whereas potato, even if higher than average, does not reach this high member count. However, the expansion within this subfamily was not uniform, and while some seem to be unique for Solanaceae (Figure 3, branch b), in other cases, the genome of Arabidopsis carries two genes whereas potato and tomato have only one.

The DRD1 subfamily tree suggests a complex evolutionary history involving a series of independent gene losses, duplication and genomic reshuffling events (recombination, transposition) resulting in a relative expansion of genes in notably tomato. It suggests that the DRD1 subfamily has gained additional functionality in tomato. The results suggest that the relative expansion has been specific for the Solanaceae, although more solanaceous genomes (*S. penelli*, *N. tabacum*, *S.* pimpinellifolium) are required to validate the specificity of this expansion for *Solanaceae* in general, or for a given species in particular.

It is supposed that the conserved ATPase domain is responsible for the energy release of DRD1 proteins, whereas other parts of the protein specify interaction partners, DNA specificity and/or sub-nuclear localization. The presence of a disordered region that may be characteristic for the expanded branch b. The differences in structure, if any, are so subtle or complex that it is difficult to associate particular sequence determinants with function. The unfolded regions occur regularly at approximately the same position in the N-terminal regions of DRD1 proteins. Such unfolded regions may help or direct protein-protein or protein-nucleic acid interactions [51–53]. Disordered regions in the DRD1 genes may therefore interface the ATPase domain to other proteins or DNA/RNA molecules [54]. This may help to specify interaction partners, whereas the lack of accessory domains indicates that ATPase-mediated remodeling is the main enzymatic function of these DRD1 subfamily members. New interaction partners could determine involvement of DRD1 proteins in new biological processes or conditions. 

Given the complex evolution and expression pattern of DRD1 genes in tomato, it is not as straightforward as for the Snf2 subfamily to transfer the function of Arabidopsis genes to the orthologous tomato genes. In Arabidopsis, several genes of this subfamily are important components of RNA-directed DNA methylation (RdDM), the pathway in which specific genomic loci are targeted for methylation by 24 bases small interfering RNAs (siRNA) [44,55]. RdDM operates in many organisms and requires common components such as DNA methyltransferases, histone modifying enzymes and RNAi proteins. 

The genes of branch a, *CLSY1* and *AtCHR42* were found in the Pol-IV polymerase protein complex [56], the RNA polymerase thought to initiate the biogenesis of the targeting siRNAs [57]. In the same complex, ATCHR31 and ATCHR40 (branch b) are also present, suggesting they play a role in the same RdDM pathway [58]. In addition to siRNAs, RdDM is also associated with the accumulation of so-called intergenic noncoding (IGN) transcripts that involves the plant specific RNA-polymerase Pol-V [59]. DRD1 (branch c) was identified in a protein complex critical for the production of Pol-V dependent IGN transcripts [56]. Recently, this gene was also established as an important player in plant immunity. Its knockout mutant showed increased susceptibility to the fungal pathogen *Plectosphaerella cucumerina* [45]. The second gene of Arabidopsis branch c, *At2g21450*, was shown to be modulated during early embryogenesis, suggesting a role after fertilization [60]. Related functions affecting small RNA accumulation and cytosine methylation have been shown for RMR1, an Snf2 ortholog in *Zea mays* (maize), in the context of paramutation [61]. As five out of six Arabidopsis DRD1 genes and RMR1 are implicated in RdDM pathways, a similar function of this subfamily in tomato is likely. 

Why tomato would need so much more active DRD1 genes than Arabidopsis? Possibly the continued selection for traits in tomato as agricultural crop has been the driving force for such developments. The functions assigned so far in Arabidopsis point in the direction of protection against biotic and abiotic stresses. The comprehensive analysis here presented shows the evolution and presence of Snf2 genes in plants. Closer evaluation of, e.g. DRD1 subfamily members, could make suitable targets for breeding and plant improvement.

## Supporting Information

Figure S1 **Schematic layout of Snf2 family ATPases.** The conserved Snf2 family ATPase region is part of the protein and consists of two Pfam domains, Snf2_N and Helicase_C, in which seven helicase motifs are present. The average size of the Snf2 family ATPase region is approx. 400aa [1]. In individual proteins, the N-terminal or C-terminal region can be very small [2].
(TIF)

Figure S2 **The number of candidate Snf2 genes in annotated plant genomes.** The total number of genes estimated for a genome is plotted above the bar in the histogram. Plant species included are organized on the basis of the position in the tree of life (shown at the left). The four species given most attention in this study (Arabidopsis, potato, tomato and *Mimulus guttatus*) are given in black.
(TIF)

Figure S3 **Full phylogenetic tree of all plant Snf2 candidates.** The tree is based on the plant data listed in table S2 and calculated with 100 bootstraps due to computational constraints. Branches with a confidence lower than 50 are marked in grey. Members not classified (n.c.) into any subfamily are indicated in light green. To increase readability, the colors of subfamily branches alternate between blue and red.
(TIF)

Figure S4 **Analysis of the Snf2 subfamily in tomato, potato, Mimulus and Arabidopsis.** The left side shows a detailed view of the DRD1 subfamily branch of an unrooted tree based on 1000 bootstraps of Snf2 data from *Arabidopsis thaliana* (Ath), *Mimulus guttatus* (Mgu), *Solanum lycopersicum* (Sly) and *Solanum tuberosum* (Stu). Confidence values (50-100) are given at the relevant branches of the tree. Identifiers give the name of the organism in three-letter abbreviations together with gene identifiers. The individual branches identified are indicated by letters in lowercase on the right side. To increase readability, some branch edges have been extended by dotted grey lines. These grey dotted lines are therefore not part of the estimated branch length. The right side shows structural elements in the protein sequence of the Snf2 subfamily members in Arabidopsis, Mimulus, tomato and potato. The individual branches identified are indicated by letters in lowercase. Besides the ATPase region, BROMO (protein-histone interaction), QLQ (protein-protein interaction) and HSA (DNA-binding) domains are present in several members. A black dot at the right end of the figure indicates the expression of the respective gene in tomato based on the analysis of RNA-seq data.
(TIF)

Figure S5 **Heat map of the RNA-seq expression data of the tomato DRD1 & Snf2 subfamily genes.** The expression is indicated as fragments per kb exon model per million mapped reads-value (FPKM-value). No cut-off was applied. Grey areas correspond to FPKM-values of 0. Gene identifiers are indicated on the x-axis with the corresponding branch name given between brackets. The biological material used to generate the RNA-seq libraries is given on the y-axis. Replicates are indicated by lowercase letters. Details on the RNA-seq libraries used are given in table S3.
(TIF)

Table S1 **Primers used for RT-PCR analysis.** The primer sequence of the forward (F) and reversed (R) primer is given for each gene identifier.
(DOC)

Table S2 **Plant data included in the analyses.** Sources are the Phytozome annotation (indicated as genome), SGN unigenes (indicated as unigene), de-novo assembled transcriptomes (indicated as transcript) and reference databases (indicated as database). The differences in Snf2 members between the annotation (first value) and the homology-based re-analysis here presented (second value) are indicated for potato (*Solanum tuberosum*).
(DOC)

Table S3 **RNA-seq libraries included in the analysis.** Data are from the short read archive (SRA; http://www.ncbi.nlm.nih.gov/sra). The library and sample IDs refer to the run and sample identifiers in SRA, respectively.
(DOC)

Dataset S1 **Text file with custom predicted gene models of *Solanum tuberosum*.**
(ZIP)

Dataset S2 **Text file of the multiple alignment of all plant Snf2 candidates.**
(ZIP)

Dataset S3 **Phylogenetic tree of all plant Snf2 candidates in NEWICK format.**
(ZIP)

References S1 (DOC)
