# Supplementary figures and images for: Snf2 Family Gene Distribution in Higher Plant Genomes Reveals DRD1 Expansion and Diversification in the Tomato Genome
Source: PLoS One. 2013 Nov 28;8(11):e81147. doi: 10.1371/journal.pone.0081147 (PMC3842944; doi:10.1371/journal.pone.0081147)

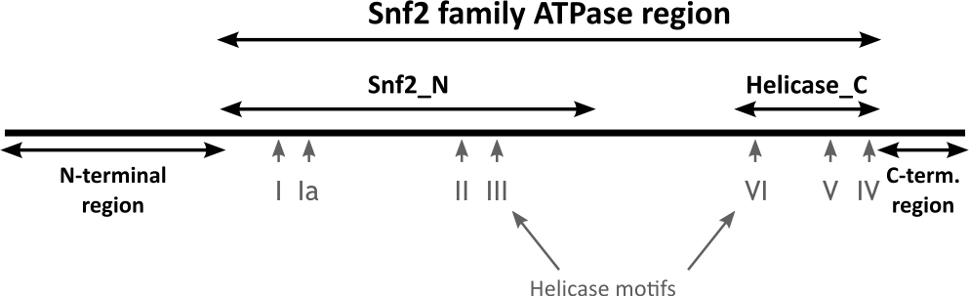

Supplement: Figure S1 — Schematic layout of Snf2 family ATPases. The conserved Snf2 family ATPase region is part of the protein and consists of two Pfam domains, Snf2_N and Helicase_C, in which seven helicase motifs are present. The average size of the Snf2 family ATPase region is approx. 400aa [1]. In individual proteins, the N-terminal or C-terminal region can be very small [2]. (TIF) [file pone.0081147.s001.tif]

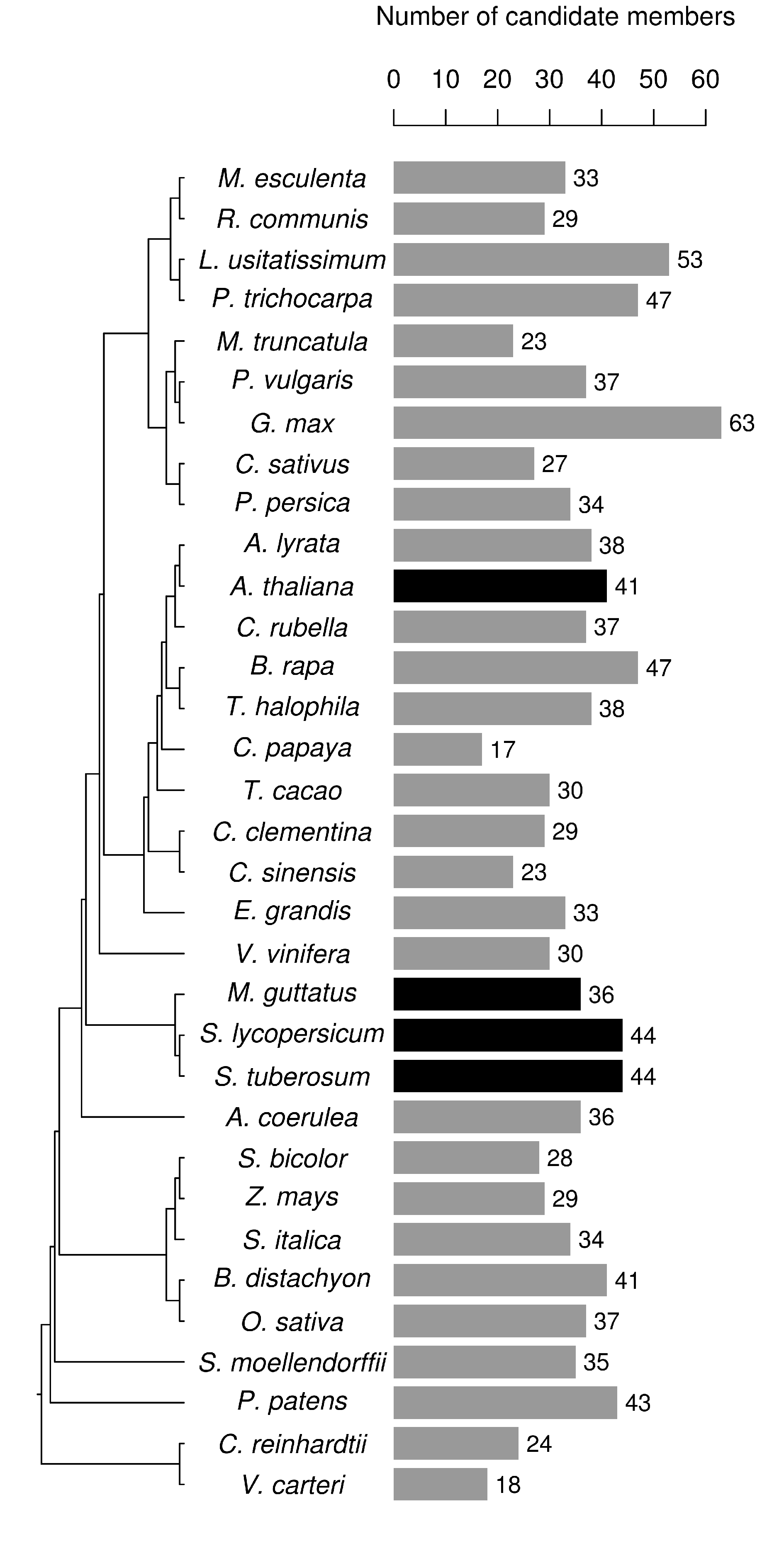

Supplement: Figure S2 — The number of candidate Snf2 genes in annotated plant genomes. The total number of genes estimated for a genome is plotted above the bar in the histogram. Plant species included are organized on the basis of the position in the tree of life (shown at the left). The four species given most attention in this study (Arabidopsis, potato, tomato and Mimulus guttatus) are given in black. (TIF) [file pone.0081147.s002.tif]

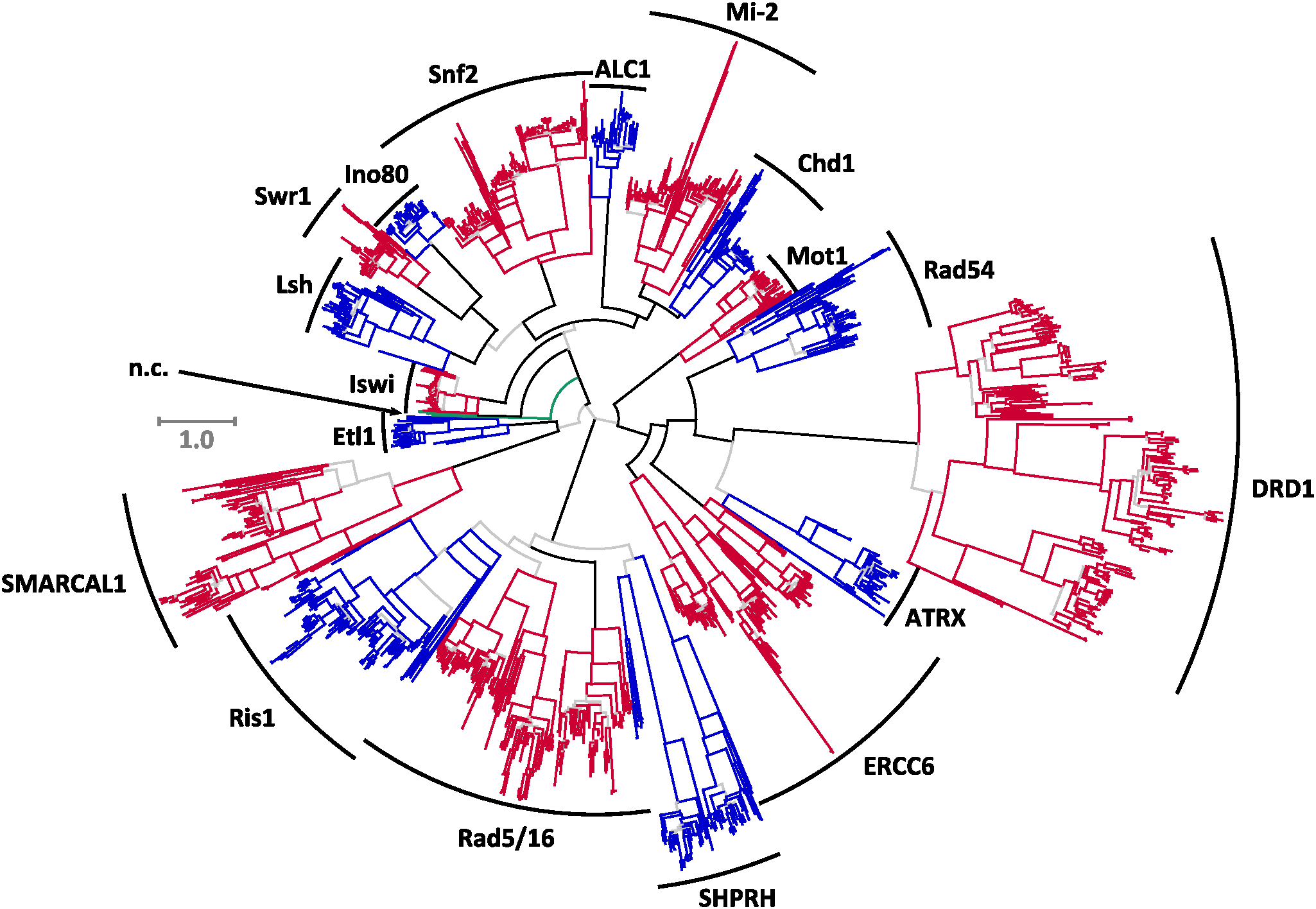

Supplement: Figure S3 — Full phylogenetic tree of all plant Snf2 candidates. The tree is based on the plant data listed in table S2 and calculated with 100 bootstraps due to computational constraints. Branches with a confidence lower than 50 are marked in grey. Members not classified (n.c.) into any subfamily are indicated in light green. To increase readability, the colors of subfamily branches alternate between blue and red. (TIF) [file pone.0081147.s003.tif]

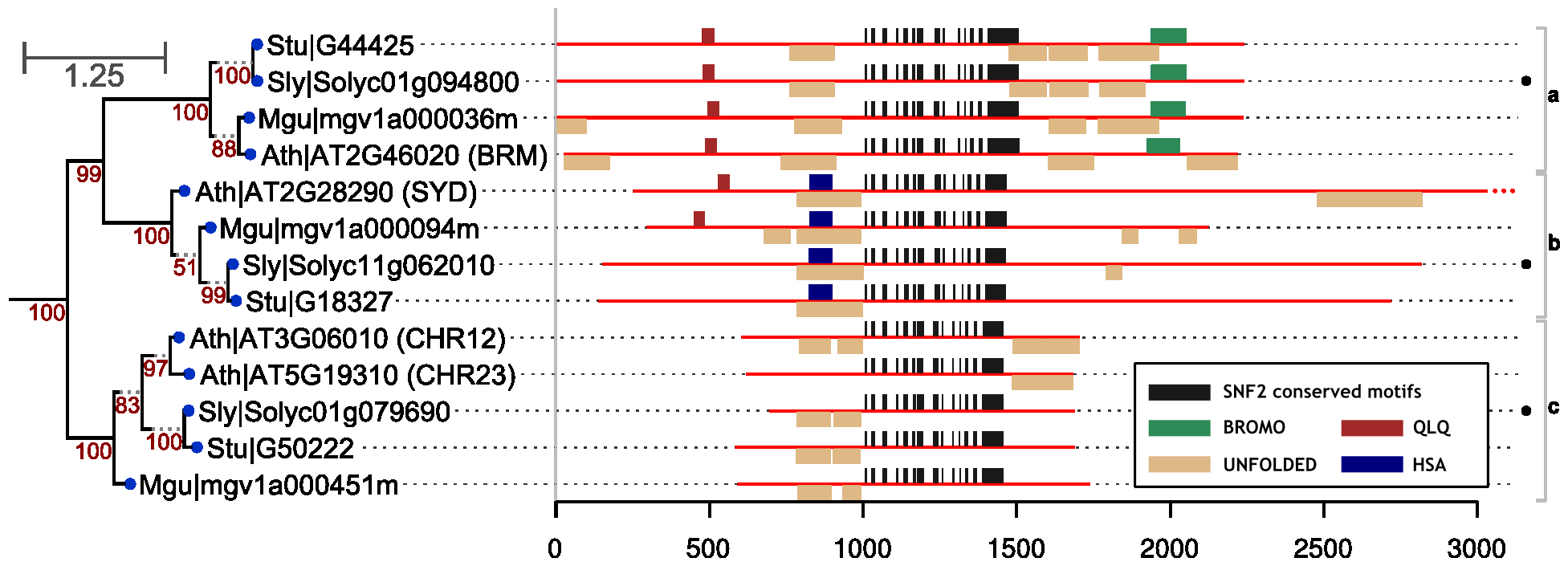

Supplement: Figure S4 — Analysis of the Snf2 subfamily in tomato, potato, Mimulus and Arabidopsis. The left side shows a detailed view of the DRD1 subfamily branch of an unrooted tree based on 1000 bootstraps of Snf2 data from Arabidopsis thaliana (Ath), Mimulus guttatus (Mgu), Solanum lycopersicum (Sly) and Solanum tuberosum (Stu). Confidence values (50-100) are given at the relevant branches of the tree. Identifiers give the name of the organism in three-letter abbreviations together with gene identifiers. The individual branches identified are indicated by letters in lowercase on the right side. To increase readability, some branch edges have been extended by dotted grey lines. These grey dotted lines are therefore not part of the estimated branch length. The right side shows structural elements in the protein sequence of the Snf2 subfamily members in Arabidopsis, Mimulus, tomato and potato. The individual branches identified are indicated by letters in lowercase. Besides the ATPase region, BROMO (protein-histone interaction), QLQ (protein-protein interaction) and HSA (DNA-binding) domains are present in several members. A black dot at the right end of the figure indicates the expression of the respective gene in tomato based on the analysis of RNA-seq data. (TIF) [file pone.0081147.s004.tif]

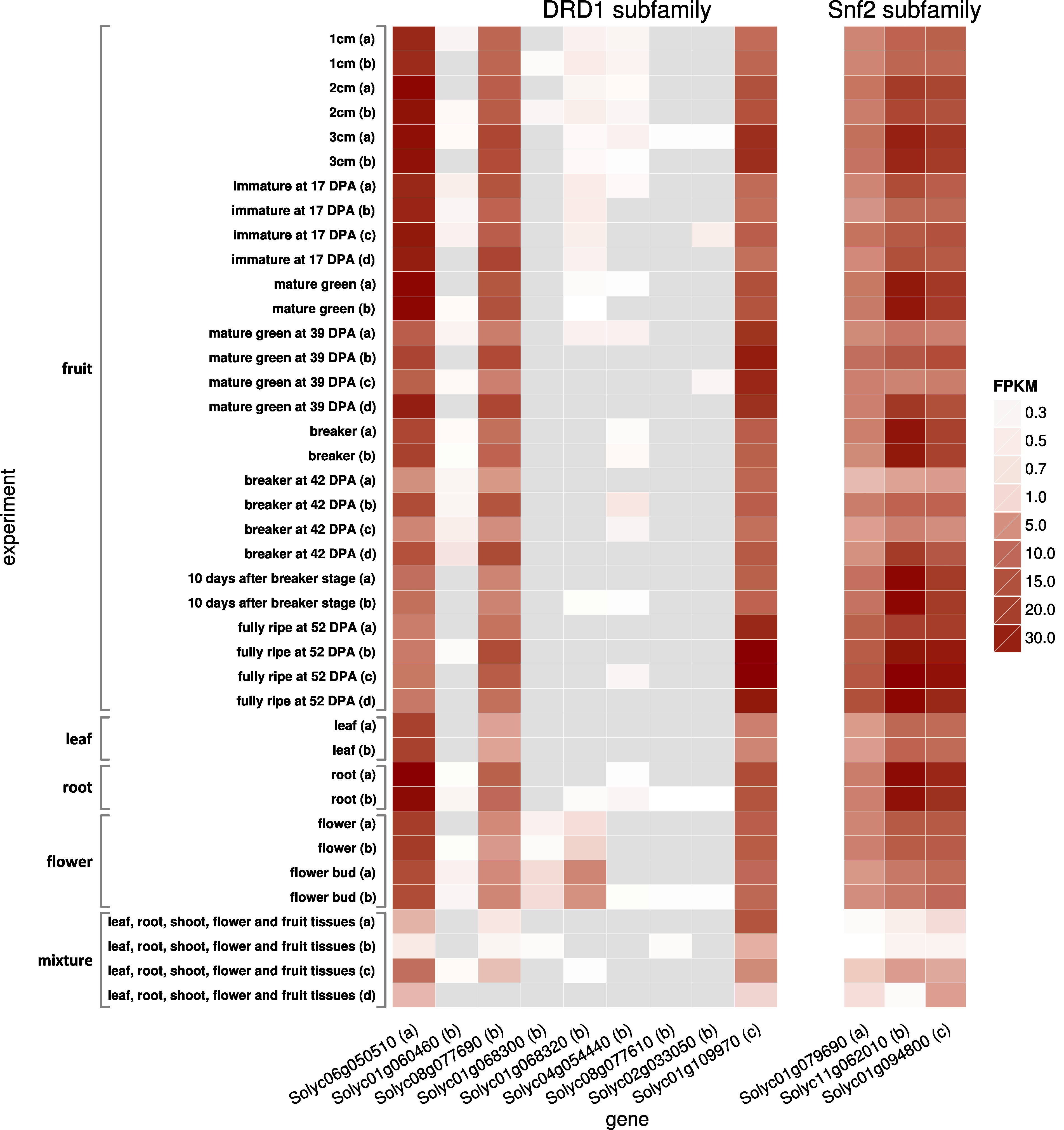

Supplement: Figure S5 — Heat map of the RNA-seq expression data of the tomato DRD1 & Snf2 subfamily genes. The expression is indicated as fragments per kb exon model per million mapped reads-value (FPKM-value). No cut-off was applied. Grey areas correspond to FPKM-values of 0. Gene identifiers are indicated on the x-axis with the corresponding branch name given between brackets. The biological material used to generate the RNA-seq libraries is given on the y-axis. Replicates are indicated by lowercase letters. Details on the RNA-seq libraries used are given in table S3. (TIF) [file pone.0081147.s005.tif]
