# Supplementary material for: Snf2 Family Gene Distribution in Higher Plant Genomes Reveals DRD1 Expansion and Diversification in the Tomato Genome
Source: PLoS One. 2013 Nov 28;8(11):e81147. doi: 10.1371/journal.pone.0081147 (PMC3842944; doi:10.1371/journal.pone.0081147)
Supplement: Table S1 — Primers used for RT-PCR analysis. The primer sequence of the forward (F) and reversed (R) primer is given for each gene identifier. (DOC) [file pone.0081147.s006.doc]

| Direction | Sequence | Gene ID |
| --- | --- | --- |
| F | GAAACAGAGAAGCGCATAGTTTT | Solyc01g068300 |
| R | GTTTTGGAGGTTGGTTACAAGAA | Solyc01g068300 |
| F | GGAAATTTAAATGACTGTCAGATGG | Solyc01g068320 |
| R | CAAGTGAATTACAGTGTCCCTTATAC | Solyc01g068320 |
| F | GAATCTATCAGTTTCGCCGATG | Solyc02g033050 |
| R | GCTTACGTTCTTTACATTTTCGCTAC | Solyc02g033050 |
| F | GAGACATAAGTGGCTGTGAGATG | Solyc04g054440 |
| R | CACTACATCTATGAACAAATGGTGA | Solyc04g054440 |
| F | CGGTGATGCAGAGTGGAG | Solyc08g077610 |
| R | GAATATCCCTAAGCTCTTCCAACG | Solyc08g077610 |
| F | GAGCAAGTACATCTTCCCTCCA | Solyc08g077690 |
| R | AGGATGAACAGAGATTAGAGACACC | Solyc08g077690 |
| F | GAAGAAGGGAAAAAGGAGTCAAA | Solyc01g060460 |
| R | TAACCATCCCCATCTTCTCC | Solyc01g060460 |
| F | CCACTTGATGTTGATGTTCCTG | Solyc06g050510 |
| R | ACCTTTTCCCTTAGAACCTCTCC | Solyc06g050510 |
| F | GGATGGACAGGAAACTAACAACA | Solyc01g109970 |
| R | CACTACCAACATTGTCACACACA | Solyc01g109970 |
| F | CCAAAATAAAAAGGAAACGCAGT | Solyc01g094800 |
| R | CCCAACTTCTCTCTATCTTTTCTTTTC | Solyc01g094800 |
| F | CTGTAATGGCGTCTCCTGCT | Solyc11g062010 |
| R | GATTTCCACTGTTGCCTCAAG | Solyc11g062010 |
| F | GTTCAGGCTTGGCATGGAA | Solyc01g079690 |
| R | CCGATAAGTGTGATGTCTCTC | Solyc01g079690 |
