# Supplementary material for: Snf2 Family Gene Distribution in Higher Plant Genomes Reveals DRD1 Expansion and Diversification in the Tomato Genome
Source: PLoS One. 2013 Nov 28;8(11):e81147. doi: 10.1371/journal.pone.0081147 (PMC3842944; doi:10.1371/journal.pone.0081147)
Supplement: Table S2 — Plant data included in the analyses. Sources are the Phytozome annotation (indicated as genome), SGN unigenes (indicated as unigene), de-novo assembled transcriptomes (indicated as transcript) and reference databases (indicated as database). The differences in Snf2 members between the annotation (first value) and the homology-based re-analysis here presented (second value) are indicated for potato (Solanum tuberosum). (DOC) [file pone.0081147.s007.doc]

| Species/Database | Genome Size (mb) | Number of predicted gene models | Number of Snf2 members | Data type | Reference |
| --- | --- | --- | --- | --- | --- |
| *Antirrhinum majus* | n/a | n/a | 0 | unigene | [3] |
| *Aquilegia coerulea* | 302 | 24823 | 36 | genome | [4] |
| *Arabidopsis lyrata* | 230 | 32670 | 38 | genome | [5] |
| *Arabidopsis thaliana* | 125 | 27416 | 41 | genome | [5] |
| *Brachypodium distachyon* | 300 | 26552 | 41 | genome | [5] |
| *Brassica rapa* | 530 | 40905 | 47 | genome | [5] |
| *Capsella rubella* | 250 | 26521 | 37 | genome | [5] |
| *Capsicum annuum* | 2700 | n/a | 0 | unigene | [3] |
| *Carica papaya* | 372 | 27769 | 17 | genome | [5] |
| *Chlamydomonas reinhardtii* | 112 | 17114 | 25 | genome | [4] |
| ChromDB (plants only) | n/a | 8618 | 377 | database | [6] |
| *Citrus clementina* | 296 | 25385 | 29 | genome | [4] |
| *Citrus sinensis* | 382 | 25379 | 23 | genome | [5] |
| *Coffea arabica* | n/a | n/a | 0 | unigene | [3] |
| *Coffea canephora* | n/a | n/a | 1 | unigene | [3] |
| *Cucumis sativus* | 367 | 21646 | 27 | genome | [5] |
| *Eucalyptus grandis* | 600 | 36376 | 33 | genome | [5] |
| *Glycine max* | 1100 | 46367 | 63 | genome | [5] |
| *Ipomoea batatas* | n/a | n/a | 0 | unigene | [3] |
| *Linum usitatissimum* | 350 | 43471 | 53 | genome | [4] |
| *Manihot esculenta* | 770 | 30666 | 33 | genome | [5] |
| *Medicago truncatula* | 500 | 50962 | 23 | genome | [5] |
| *Mimulus guttatus* | 430 | 26718 | 36 | genome | [5] |
| *Nicotiana benthamiana* | n/a | n/a | 0 | unigene | [3] |
| *Nicotiana sylvestris* | n/a | n/a | 0 | unigene | [3] |
| *Nicotiana tabacum* | n/a | n/a | 4 | unigene | [3] |
| *Oryza sativa* | 433 | 55986 | 37 | genome | [5] |
| *Petunia hybrid cultivar* | n/a | n/a | 0 | unigene | [3] |
| *Phaseolus vulgaris* | 487 | 26374 | 37 | genome | [4] |
| *Physcomitrella patens* | 480 | 32273 | 43 | genome | [4] |
| *Populus trichocarpa* | 485 | 40668 | 47 | genome | [5] |
| *Prunus persica* | 220 | 27864 | 34 | genome | [5] |
| RefSeq (plants only) | n/a | 519211 | 195 | database | [7] |
| *Ricinus communis* | 400 | 31221 | 29 | genome | [5] |
| *Selaginella moellendorffii* | 213 | 22285 | 35 | genome | [4] |
| *Setaria italica* | 515 | 35471 | 34 | genome | [5] |
| *Solanum dulcamara* | n/a | 14288 | 12 | transcript | [3] |
| *Solanum lycopersicum* | 900 | 34727 | 44 | genome | [8] |
| *Solanum melongena* | 1100 | n/a | 0 | unigene | [3] |
| *Solanum peruvianum* | n/a | 17280 | 34 | transcript | [3] |
| *Solanum tuberosum* | 840 | 39031 | 23/44 | genome | [5] |
| *Sorghum bicolor* | 770 | 27608 | 28 | genome | [5] |
| *Thellungiella halophila* | 243 | 26351 | 38 | genome | [4] |
| *Theobroma cacao* | 430 | 46143 | 30 | genome | [9] |
| UniRef100 (plants only) | n/a | 591965 | 46 | database | [10] |
| *Vitis vinifera* | 475 | 26346 | 30 | genome | [11] |
| *Volvox carteri* | 131 | 14971 | 18 | genome | [4] |
| *Zea mays* | 2500 | 39656 | 29 | genome | [5] |
