# Supplementary material for: Snf2 Family Gene Distribution in Higher Plant Genomes Reveals DRD1 Expansion and Diversification in the Tomato Genome
Source: PLoS One. 2013 Nov 28;8(11):e81147. doi: 10.1371/journal.pone.0081147 (PMC3842944; doi:10.1371/journal.pone.0081147)
Supplement: Table S3 — RNA-seq libraries included in the analysis. Data are from the short read archive (SRA; http://www.ncbi.nlm.nih.gov/sra). The library and sample IDs refer to the run and sample identifiers in SRA, respectively. (DOC) [file pone.0081147.s008.doc]

| Name | Library ID | Sample ID |
| --- | --- | --- |
| 1cm (a) | SRR404317 | SRS291272 |
| 1cm (b) | SRR404318 | SRS291272 |
| 2cm (a) | SRR404319 | SRS291273 |
| 2cm (b) | SRR404320 | SRS291273 |
| 3cm (a) | SRR404321 | SRS291274 |
| 3cm (b) | SRR404322 | SRS291274 |
| immature at 17 DPA (a) | SRR346617 | SRS265321 |
| immature at 17 DPA (b) | SRR346618 | SRS265321 |
| immature at 17 DPA (c) | SRR346619 | SRS265321 |
| immature at 17 DPA (d) | SRR346620 | SRS265321 |
| mature green (a) | SRR404324 | SRS291275 |
| mature green (b) | SRR404325 | SRS291275 |
| mature green at 39 DPA (a) | SRR346621 | SRS265322 |
| mature green at 39 DPA (b) | SRR346622 | SRS265322 |
| mature green at 39 DPA (c) | SRR346623 | SRS265322 |
| mature green at 39 DPA (d) | SRR346624 | SRS265322 |
| breaker (a) | SRR404326 | SRS291276 |
| breaker (b) | SRR404327 | SRS291276 |
| breaker at 42 DPA (a) | SRR346625 | SRS265323 |
| breaker at 42 DPA (b) | SRR346626 | SRS265323 |
| breaker at 42 DPA (c) | SRR346627 | SRS265323 |
| breaker at 42 DPA (d) | SRR346628 | SRS265323 |
| 10 days after breaker stage (a) | SRR404328 | SRS291277 |
| 10 days after breaker stage (b) | SRR404329 | SRS291277 |
| fully ripe at 52 DPA (a) | SRR346629 | SRS265324 |
| fully ripe at 52 DPA (b) | SRR346630 | SRS265324 |
| fully ripe at 52 DPA (c) | SRR346631 | SRS265324 |
| fully ripe at 52 DPA (d) | SRR346632 | SRS265324 |
| leaf (a) | SRR404309 | SRS291268 |
| leaf (b) | SRR404310 | SRS291268 |
| root (a) | SRR404311 | SRS291269 |
| root (b) | SRR404312 | SRS291269 |
| flower (a) | SRR404313 | SRS291270 |
| flower (b) | SRR404314 | SRS291270 |
| flower bud (a) | SRR404315 | SRS291271 |
| flower bud (b) | SRR404316 | SRS291271 |
| leaf, root, shoot, flower and fruit tissues (a) | SRR346633 | SRS265325 |
| leaf, root, shoot, flower and fruit tissues (b) | SRR346634 | SRS265325 |
| leaf, root, shoot, flower and fruit tissues (c) | SRR346635 | SRS265325 |
| leaf, root, shoot, flower and fruit tissues (d) | SRR346636 | SRS265325 |
