# Supplementary material for: Snf2 Family Gene Distribution in Higher Plant Genomes Reveals DRD1 Expansion and Diversification in the Tomato Genome
Source: PLoS One. 2013 Nov 28;8(11):e81147. doi: 10.1371/journal.pone.0081147 (PMC3842944; doi:10.1371/journal.pone.0081147)
Supplement: References S1 — (DOC) [file pone.0081147.s012.doc]

**Supporting References**

1. Eisen JA, Sweder KS, Hanawalt PC (1995) Evolution of the SNF2 family of proteins: subfamilies with distinct sequences and functions. Nucleic Acids Res 23: 2715–2723.

2. Flaus A, Owen-Hughes T (2011) Mechanisms for ATP-dependent chromatin remodeling: the means to the end. FEBS J 278: 3579–3595. doi:10.1111/j.1742-4658.2011.08281.x.

3. Bombarely A, Menda N, Tecle IY, Buels RM, Strickler S, et al. (2011) The Sol Genomics Network (solgenomics.net): growing tomatoes using Perl. Nucleic Acids Res 39: D1149–1155. doi:10.1093/nar/gkq866.

4. Goodstein DM, Shu S, Howson R, Neupane R, Hayes RD, et al. (2012) Phytozome: a comparative platform for green plant genomics. Nucleic Acids Res 40: D1178–1186. doi:10.1093/nar/gkr944.

5. Feuillet C, Leach JE, Rogers J, Schnable PS, Eversole K (2010) Crop genome sequencing: lessons and rationales. Trends Plant Sci 16: 77–88. doi:10.1016/j.tplants.2010.10.005.

6. Gendler K, Paulsen T, Napoli C (2008) ChromDB: the chromatin database. Nucleic Acids Res 36: D298–302. doi:10.1093/nar/gkm768.

7. Pruitt KD, Tatusova T, Brown GR, Maglott DR (2012) NCBI Reference Sequences (RefSeq): current status, new features and genome annotation policy. Nucleic Acids Res 40: D130–135. doi:10.1093/nar/gkr1079.

8. Sato S, Tabata S, Hirakawa H, Asamizu E, Shirasawa K, et al. (2012) The tomato genome sequence provides insights into fleshy fruit evolution. Nature 485: 635–641. doi:10.1038/nature11119.

9. Argout X, Salse J, Aury J-M, Guiltinan MJ, Droc G, et al. (2011) The genome of *Theobroma cacao*. Nat Genet 43: 101–108. doi:10.1038/ng.736.

10. Suzek BE, Huang H, McGarvey P, Mazumder R, Wu CH (2007) UniRef: comprehensive and non-redundant UniProt reference clusters. Bioinformatics 23: 1282–1288. doi:10.1093/bioinformatics/btm098.

11. Jaillon O, Aury J-M, Noel B, Policriti A, Clepet C, et al. (2007) The grapevine genome sequence suggests ancestral hexaploidization in major angiosperm phyla. Nature 449: 463–467. doi:10.1038/nature06148.
